# Inter and intra-reliability of ultrasonography for the measurement of abdominal subcutaneous & visceral adipose tissue thickness at 12 weeks gestation

**DOI:** 10.1186/s12880-019-0393-6

**Published:** 2019-12-17

**Authors:** Alexandra Cremona, Kevin Hayes, Clodagh S. O’Gorman, Ciara Ní Laighin, Khadijah I. Ismail, Alan E. Donnelly, Jill Hamilton, Amanda Cotter

**Affiliations:** 1 Graduate Entry Medical School (GEMS), University of Limerick, Limerick, Ireland; 2 School of Allied Health (SAH), University of Limerick, Limerick, Ireland; 3 Institute of Nutrition & Dietetics (INDI), Dublin, Ireland; 4 Health Research Institute (HRI), University of Limerick, Limerick, Ireland; 5 Physical Education and Sport Sciences (PESS), University of Limerick, Limerick, Ireland; 6 Department of Statistics, University College Cork (UCC), Cork, Ireland; 7 University Maternity Hospital Limerick (UMHL), Limerick, Ireland; 8 The Hospital for Sick Children, Toronto, Canada

**Keywords:** Adipose, Visceral, Reliability, Ultrasound, Technique, Pregnancy, Subcutaneous, Body composition

## Abstract

**Background:**

Excess abdominal adiposity cause metabolic disturbances, particularly in pregnancy. Methods of accurate measurement are limited in pregnancy due to risks associated with these procedures. This study outlines a non-invasive methodology for the measurement of adipose tissue in pregnancy and determines the intra- and inter-observer reliability of ultrasound (US) measurements of the two components of adipose tissue (subcutaneous (SAT) and visceral adipose tissue (VAT)) within a pregnant population.

**Methods:**

Thirty pregnant women were recruited at the end of their first trimester, from routine antenatal clinic at the University Maternity Hospital Limerick, Ireland. Measurements of adipose tissue thickness were obtained using a GE Voluson E8 employing a 1–5 MHz curvilinear array transducer. Two observers, employing methodological rigour in US technique, measured thickness of adipose tissue three times, and segmented the US image systematically in order to define measurements of SAT and VAT using specifically pre-defined anatomical landmarks.

**Results:**

Intra-observer and inter-observer precision was assessed using Coefficient of Variation (CV). Measurements of SAT and total adipose for both observers were < 5% CV and < 10% CV for VAT in measures by both observers. Inter-observer reliability was assessed by Limits of Agreement (LoA). LoA were determined to be − 0.45 to 0.46 cm for SAT and − 0.34 to 0.53 cm for VAT values. Systematic bias of SAT measurement was 0.01 cm and 0.10 cm for VAT. Inter-observer precision was also assessed by coefficient of variation (CV: SAT, 3.1%; VAT, 7.2%; Total adipose, 3.0%).

**Conclusion:**

Intra-observer precision was found to be acceptable for measures of SAT, VAT and total adipose according to anthropometric criterion, with higher precision reported in SAT values than in VAT. Inter-observer reliability assessed by Limits-Of-Agreement (LoA) confirm anthropometrically reliable to 0.5 cm. Systematic bias was minimal for both measures, falling within 95% confidence intervals. These results suggest that US can produce reliable, repeatable and accurate measures of SAT and VAT during pregnancy.

## Background

Ultrasound (US) has been used effectively to assess body fat for decades [1]. Limitations to its use are due to lack of standardization of technique, and data on repeatability amongst different operators [2, 3]. The current gold standard for the quantitative assessment of intra-abdominal adipose tissue uses computed tomography (CT) scanning [4]. Validity and reproducibility of ultrasound techniques against CT scanning has been previously assessed [5–8] in non-pregnant populations, and reportedly the inter-observer correlation coefficient of the mean ultrasound distance was 0.94 (*P* < 0.001), and coefficient of variation 5.4% within a non-pregnant population [7]. Other methods for quantifying risk using abdominal measures and ratios of these, are; DXA scanning, waist: hip circumference ratio, and anthropometric skinfold measurements. However, during pregnancy these three techniques have distinct disadvantages, which render them inadequate within a clinical setting and in a pregnant population [9, 10]. Limitations include exposure to ionising radiation, expense, lack of validation of technique, time-consuming techniques and requirement of a trained skilful measurer [9, 10].

Despite methods of capturing body composition being limited within a pregnant population, the use of ultrasound to measure abdominal adipose tissue has been recently reviewed and found to be a useful tool for measuring body composition non-invasively [2, 3]. While US requires skill and training with a cost implication, pregnant women undergo US by a skilled ultra-sonographer at the end of the first trimester, as part of routine care, making this a contact point with healthcare professionals with potential opportunity for measurements to be carried out. Measuring components of abdominal adipose tissue- visceral adipose tissue (VAT) and subcutaneous adipose tissue (SAT), are of particular current relevance and importance as these depots of adiposity have been implicated in the pathogenesis of metabolic and cardiovascular health in non-pregnant populations [3, 11, 12], as well as in a pregnant population [13].

Maternal obesity has been linked to increased morbidity and mortality in pregnancy putting both the mother and infant at risk in the short and long term [14, 15]. Large population studies looking at pregnancy outcomes based on the World Health Organisation (WHO) body mass index (BMI) sub-classifications of obesity [16] found a relationship to increasing risk of adverse outcomes, including gestational diabetes, hypertensive disorders, caesarean section, macrosomia, admission to neonatal unit and neonatal hypoglycaemia [15, 17, 18]. However, BMI does not provide insight into components of body composition, such as lean tissue, subcutaneous or visceral adipose tissue which are known to exert different physiological effects in the pregnancy state [19]. Crude measures of adipose thickness such as that possible via ultrasound, provide a non-invasive technique for insight into subcutaneous and visceral adipose compartments of body composition. It is understood that visceral fat, specifically pre-peritoneal fat thickness, has been identified in the production of excess adipokines which play a role in increased insulin resistance by disrupting post-insulin signalling mechanisms, thus contributing to the pathogensis of gestational diabetes mellitus [20, 21]. It has also been associated with an increase in other cardio-metabolic risk factors within various studies [12, 22–24]. The role of subcutaneous fat in the development of obesity related disorders remains controversial according to a recent review by Bazzocchi, et al. [3]. The review attributes contradictory findings from investigations of subcutaneous fat [25, 26], to the variation in location of the measurement and lack of consistency in the methods used to capture this specific depot of adipose [27].

To be clinically useful within a pregnant population, reliability and reproducibility of abdominal fat quantification needed to be assessed within this specific population. Therefore, this study sought to standardise and outline a technically rigorous methodology used to quantify abdominal adipose tissue in pregnant women, and to segment this into its constituents, visceral (VAT) measured as the pre-peritoneal fat thickness, and subcutaneous adipose tissue (SAT) as the minimum abdominal subcutaneous fat thickness. Subsequently, both inter- and intra-observer variability were assessed in order to test the reliability of these measures in a pregnant population.

## Methods

### Study population

Thirty subjects were recruited prospectively. These subjects were attending the University Maternity Hospital Limerick (Ireland) for their first routine antenatal visit at 12-weeks gestation, at which an ultrasound scan is routinely performed. Informed consent was sought and granted (REC 082/17) in accordance with the ethical recommendations of Health Service Executive (HSE) Mid Western Hospital Research Ethics Committee.

### Ultrasonography

Measurements of adipose tissue were taken via abdominal ultrasonography (US) using a GE Voluson E8 employing a 1–5 MHz curvilinear array transducer. This transducer was a practical choice as it required no changeover from the preceding obstetric scan, and the frequency was sufficiently high to provide adequate resolution at the shallow depth of measurement.

**Fig. 1:**
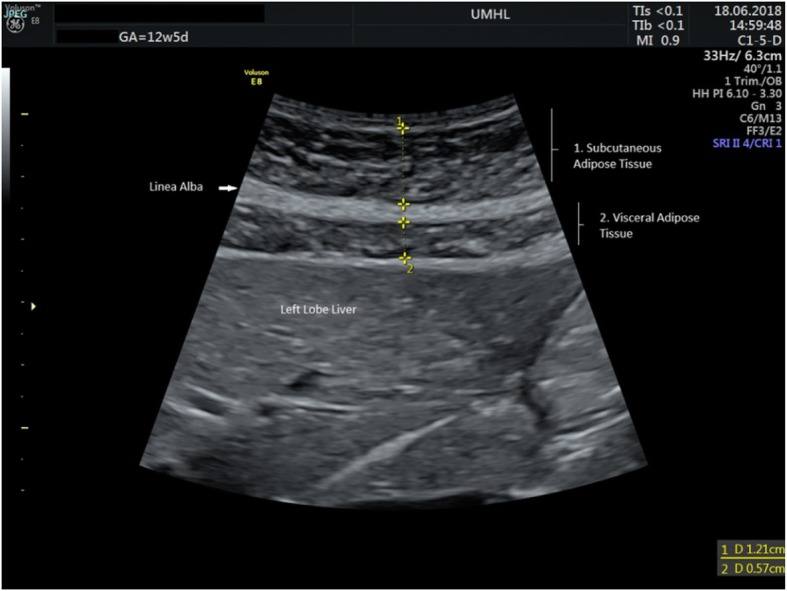
Example ultrasound screenshot image at correct position for measurement of SAT (1) and VAT (2)

**Fig. 2:**
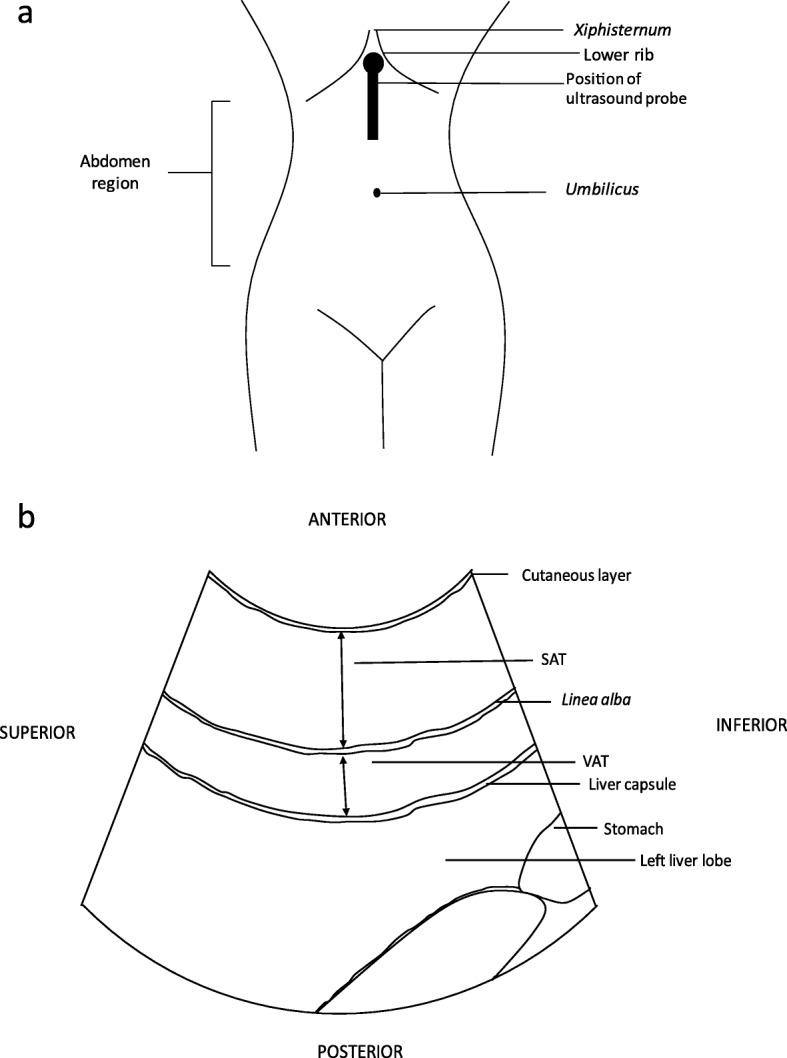
**a** Anatomical schematic representation of *xiphisternum* in relation to positioning of ultrasound probe. **b** Anatomical schematic of ultrasound image to illustrate anatomical landmarks and positioning. SAT Subcutaneous Adipose Tissue; VAT Visceral Adipose Tissue (pre-peritoneal fat)

With the patient in a supine position and the transducer perpendicular to the skin, the required image was obtained in sagittal plane at the *xiphisternum,* producing a longitudinal view of the left lobe of liver and the aorta (see Fig. 1). Minimal pressure was exerted on the skin, in order to avoid compression of the adipose tissue. The transducer was rocked left to right, in order to identify the narrowest projection of the *linea alba*. The scan depth was reduced; excluding the aorta from the image. The sector width was reduced to 40 degrees; increasing line density. Thus, an image of both layers of adipose tissue was obtained with the inferior part of the left lobe of liver seen posteriorly (see Fig. 2a and b). At this point, the time gain compensation (TGC) and overall gain were adjusted carefully to allow clear visualisation of subcutaneous adipose tissue and homogenous echogenicity within the left lobe of liver. The image was then frozen.

Calipers were placed to measure in millimetres. Subcutaneous fatty tissue was measured from the lower border of the cutaneous layer to the upper border of the *linea alba* and visceral fatty tissue was measured from the lower border of *linea alba* to the upper border of the liver capsule.

The US measurements were performed at the time of routine first trimester ultrasound examination. Both observers took 3 measurements each of both VAT and SAT on 30 subjects. A new image was acquired between each set of measurements. The second observer entered the examination room once the first observer’s measurements were completed and removed from the screen. All six images were saved using ViewPoint™, GE’s ultrasound image management and reporting solution software and were identified with each observers’ initials prior to transfer. Measurements recorded by observer 1 where undertaken by an obstetrician, and measurements by observer 2 where undertaken by a trained radiographer. Both observers were regularly involved in antenatal scanning at this clinical site.

### Statistical methods

SAT and VAT on 30 subjects were obtained by two observers, (coded here as K and C). Each measurement was replicated 3 times, (with the exception of one occasion where observer K made just two replicate measurements of subcutaneous fat on a particular subject).

**Fig. 3:**
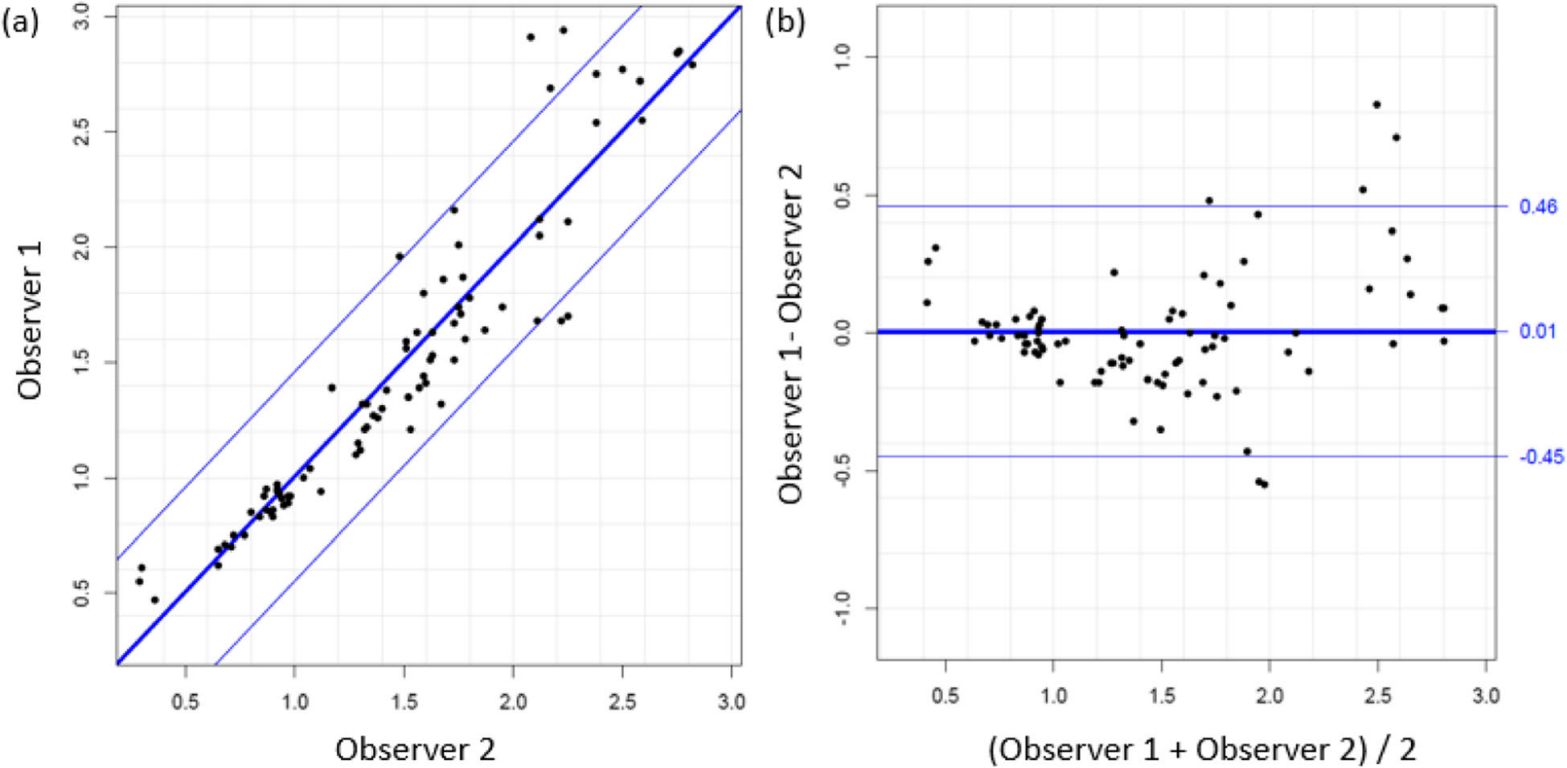
**a** & **b** Graphical comparisons of the measurements obtained for SAT by the two observers were made using Bland-Altman plots showing unlinked replicates (**a**). On the right; Plot of difference between measures of observer 1 and observer 2 against the mean of the two measurements of SAT (**b**). Solid line represents the mean; upper line shows the mean + 1.96 SD and lower line the mean − 1.96 SD

**Fig. 4:**
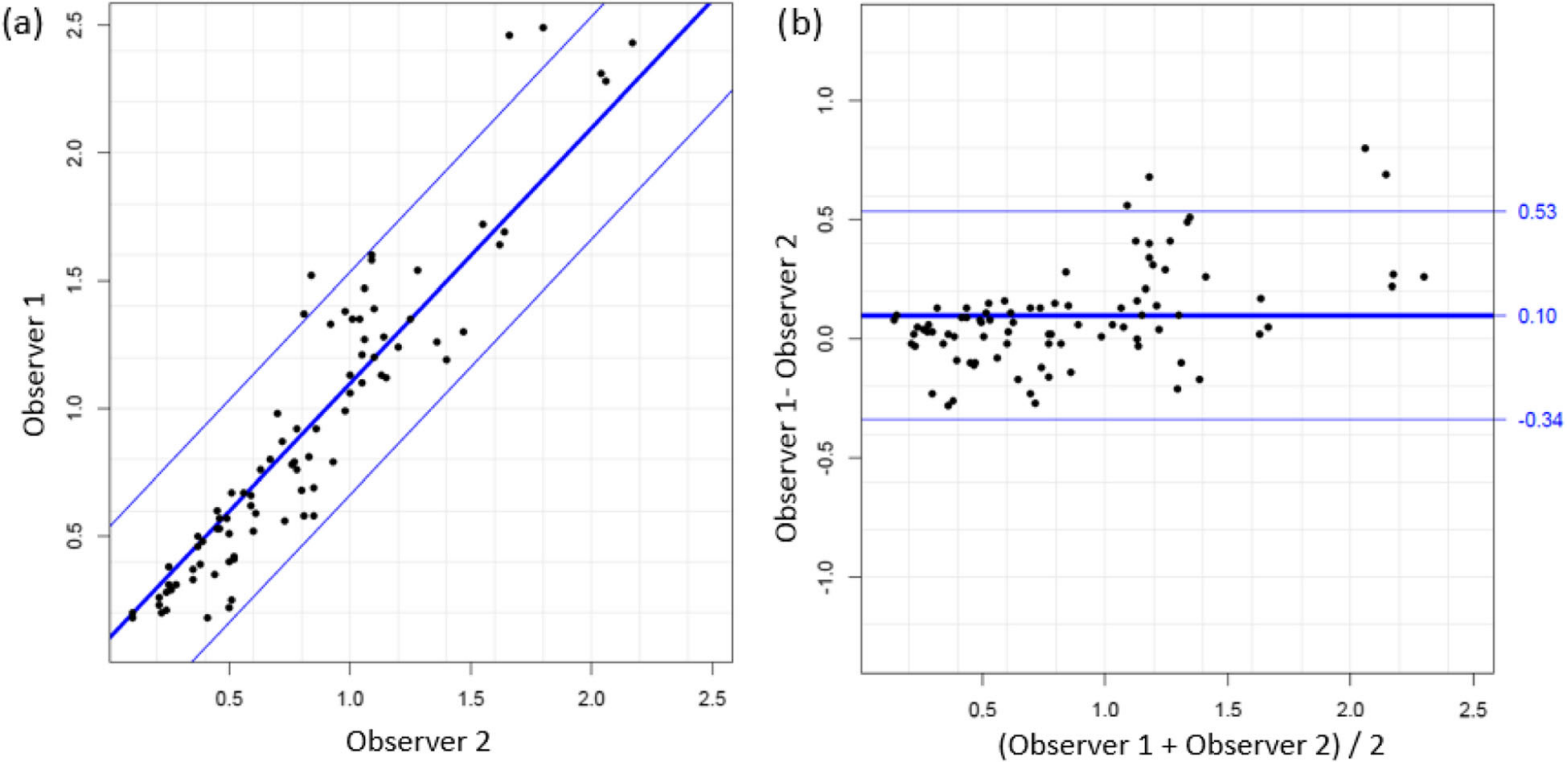
**a** & **b** Graphical comparisons of the measurements obtained for VAT by the two observers were made using Bland-Altman plots showing unlinked replicates (**a**). On the right; Plot of difference between measures of observer 1 and observer 2 against the mean of the two measurements of VAT (**b**). Solid line represents the mean; upper line shows the mean + 1.96 SD and lower line the mean − 1.96 SD

Intra-observer precision was assessed using Coefficient-of-Variation (Table 1). Inter-observer reliability was assessed using Coefficient-of-variation, and precision expressed as repeatability standard deviations (SD) and their respective coefficients, as well as Limits-of-Agreement. Graphical comparisons of the measurements obtained by the two observers were made using Bland-Altman plots showing unlinked replicates [28]. These were generated using the method comparison studies *R* package *MethComp* [29] which determines the LoA by fitting a variance component model that assumes unlinked replicates [30]. Comparison plots were constructed (see Figs. 3a, b and 4a, b). Estimates of the LoA were obtained from the corresponding variance component model.

**Table 1:** Coefficient of variation (CV) for observer 1 and observer 2 based on triplicate measures of SAT, VAT and total adipose (SAT + VAT) on replicates (*n* = 30)

	Observer 1		Observer 2	
Thickness (cm)	Mean (±SD)	%CV	Mean (±SD)	%CV
SAT	1.5 ± 0.1	2.6	1.5 ± 0.1	3.5
VAT	1.0 ± 0.1	5.8	0.9 ± 0.1	8.5
Total adipose	2.5 ± 0.1	2.6	2.4 ± 0.1	3.3

## Results

Intra-observer precision was assessed by co-efficient of variation. The data are presented in Table 1. Intra-observer precision was to an acceptable degree according to anthropometric criterion. Measurements of SAT and total adipose for both observers was < 5% CV and level for VAT < 10% CV for both observers.

Inter-observer precision and repeatability was also assessed by co-efficient of variation, with %CV of 3.05, 7.15 and 2.95% for SAT, VAT and total adipose respectively. Inter-observer repeatability standard deviations and coefficients are presented in Table 2.

**Table 2:** Inter-observer repeatability SDs and repeatability coefficients (CV). SD: standard deviation of the difference between two measurements by the same method on the item under identical circumstances; the repeatability coefficient the numerical extent of the prediction interval for this difference, i.e. 2*sqrt (2) *SD

	SAT		VAT		Total adipose	
	SD	CV	SD	CV	SD	CV
Observer 1	0.079	0.157	0.087	0.174	0.122	0.245
Observer 2	0.107	0.215	0.128	0.256	0.155	0.310

Inter-observer reliability was also assessed by LoA. Determinations by each of the two observers was made in triplicate. LoA were determined to be − 0.45 to 0.46 cm for SAT and − 0.34 to 0.53 cm for VAT values. Systematic bias of SAT measurement was 0.01 cm and VAT, 0.10 cm (*p* > 0.05).

## Discussion

The results of this reliability study show that intra-abdominal ultrasound, using a strict protocol, is a reliable method to assess the amount of subcutaneous and visceral adipose tissue. Within the same operator, intra-observer precision is acceptable for measures of SAT, VAT and total adipose, with higher precision in SAT values than VAT. Between different operators, inter-observer reliability assessed by LoA confirm anthropometrically reliable to 0.5 cm. Systematic bias was minimal for both measures falling within 95% confidence intervals.

Adipose tissue has been previously measured via various specific methodologies, to produce different indices including intra-abdominal fat, abdominal wall fat index, pre-peritoneal fat, mesenteric fat and several others. These have been defined and characterised based on the specific anatomical sites utilized to measure them, as well as specific conditions such as fasting and breathe exhalation. As well as characterizing these methodologies, Bazzocchi *et al., *[3] compiles the work around the validation of these measures via ultrasound technique against the gold standard CT scanning and their reproducibility (intra -observer and inter- observer reliability). Pre-peritoneal fat thickness, (defined as the measurement taken on xiphoumbilical line just below the xiphoid process, as the major distance between the anterior surface of the peritoneum covering the liver lobe, to the posterior surface of the *linea alba*) is the specific abdominal adipose index measured in this study, and has been validated with success against CT imaging in recent times [8], demonstrated strong correlation between the CT imaging and US techniques (Lin’s correlation coefficient of 0.85–0.87). Minimum Subcutaneous fat thickness (determined as the distance between the anterior surface of the *linea alba* and the peritoneum covering the liver lobe, in the same anatomical place of maximum preperitoneal fat thickness) also had excellent correlation with CT imaging (Lin’s correlation coefficient of 0.94–0.96). Inter and intra-observer reliability has also been found to be very acceptable in obese and non-obese patients, with coefficients of variation reported between 4.3 and 6.4% [8, 12]. Reliability of these measures has never been tested in a pregnant population, and this is important as hydration of tissue changes during pregnancy, affects compressibility of tissues and therefore potentially introducing a source of error whilst undertaking measurements [31, 32]. Thus, this study contributes to this area of research in a population where the obesity epidemic is pertinent and timely.

The link between metabolic health and adiposity in pregnancy is currently a fertile ground of research [33–36]. Excessive accumulation of adipose tissue into the viscera, has been implicated in increased risk of cardio-metabolic risk [13, 37, 38] and diabetes mellitus [13, 38–40]. Further to this, some studies have investigated measures of abdominal adipose tissue in early pregnancy, and established its ability to predict glucose intolerance and gestational diabetes in later pregnancy [41–46]. These research investigations give insight into how measures in early pregnancy can play an important role in earlier diagnosis and/or intervention, at a time when there is established contact with healthcare professionals [47]. Further to this, early detection of risk or diagnosis of gestational diabetes in pregnancy has been found to be critical in improving outcomes of various types of interventions, with dietary [48]; exercise [49]; pharmacological [50] intervention, as well a combination of these [51], showing improved outcomes when applied for a longer time-span.

The end of the first trimester (12 weeks gestation) is a clinically significant clinical time-point, at which women attend routine antenatal appointments to the maternity hospital to undertake an ultrasound scan to determine gestation, foetal number and to out-rule major foetal abnormalities. Following the scan the patient is booked in the Antenatal Clinic; medical and pregnancy history and any comorbidities are recorded and antenatal bloods are drawn. This ‘booking visit’ therefore presents a special opportunity of contact with healthcare staff which should be utilized effectively and efficiently to identify women at higher risk in order to improve management of disease.

However, this specific population is difficult to research with respect to obtaining ethical approval and consent due to concerns of invasive procedures during such a  vulnerable period. The proposed US method to measure abdominal adipose is safe, non-invasive, economical, and does not involve any extra intervention for the patient- such as, specific procedural preparation. In addition, it is time-efficient, as it took the researchers less than 3 minutes to record three repeated measures on one person. We would recommend that future studies incorporate recording time taken to take the measurements. In practice, routine scans at antenatal visit are allocated 15 min each, despite variations according to clinical requirement. Future studies examining relationships between abdominal adiposity and pregnancy outcomes may prove to be clinically useful in terms of risk- stratification, therefore using US as a research tool will translate easily to an applicable tool in practice. Currently, body mass index is used as a risk-stratification tool during pregnancy; however, this does not capture body composition parameters such as abdominal adiposity, which are specifically implicated in metabolic health [11, 12, 20, 21].

## Conclusions

Ultrasound is a non-invasive, safe, quick and available tool for quantifying adiposity in both clinical practice as a research tool. Standardized techniques for abdominal adiposity, specifically visceral fat thickness at the xiphoid region (pre-peritoneal fat) has not been previously investigated in a pregnant population, thus addressing a gap within the literature previously identified [2, 3]. Therefore, this study offers a technique which when replicated, is a highly reliable and practical tool, which does not require demanding operator training and can therefore be implemented by researchers and clinicians during routine antenatal ultrasonography. Measurement of intra-abdominal adipose by ultrasound is suitable for use in prospective observational or interventional studies in pregnant women.

## Data Availability

Data is available and can be accessed online in the supplementary material.

## Abbreviations

BMI: Body mass index
CT: Computed Tomography
CV: Coefficient of variation
DXA: Dual-energy X-ray absorptiometry
LoA: Limits of agreement
SAT: Subcutaneous adipose tissue
TGC: Time gain compensation
US: Ultrasound
VAT: Visceral adipose tissue
WHO: World Health Organisation
